# Mpox Vaccine Acceptance, Democratic Republic of the Congo

**DOI:** 10.3201/eid3012.241226

**Published:** 2024-12

**Authors:** Skylar Petrichko, Jason Kindrachuk, Dalau Nkamba, Megan Halbrook, Sydney Merritt, Handdy Kalengi, Leonard Kamba, Michael Beya, Nicole A. Hoff, Christophe Luhata, Didine K. Kaba, Anne W. Rimoin

**Affiliations:** Jonathan and Karin Fielding School of Public Health, University of California, Los Angeles, California, USA (S. Petrichko, M. Halbrook, S. Merritt, N.A. Hoff, A.W. Rimoin); Max Rady College of Medicine, University of Manitoba, Winnipeg, Manitoba, Canada (J. Kindrachuk); Kinshasa School of Public Health, University of Kinshasa, Kinshasa, Democratic Republic of the Congo (D. Nkamba, H. Kalengi, L. Kamba, M. Beya, D.K. Kaba); Ministry of Health, Kinshasa (C. Luhata)

**Keywords:** mpox, MPXV, vaccination, viruses, zoonoses, Democratic Republic of the Congo

## Abstract

We report general acceptance (61.0%) of an mpox vaccine in the Democratic Republic of the Congo among 5,226 survey respondents. Healthcare workers and respondents in historic mpox-endemic regions had higher acceptance rates. These data highlight the need for increased community engagement and sensitization before widespread deployment of mpox vaccines.

Mpox, caused by monkeypox virus (MPXV), is a zoonotic infectious disease endemic to the Democratic Republic of the Congo (DRC) (*1*–*3*). In 2022, rapid worldwide spread of MPXV clade IIb resulted in >91,000 confirmed infections and prompted the World Health Organization to declare a public health emergency of international concern (*4*). In DRC, incident cases quadrupled from 2021 to 2023; during January 2023–July 2024, >28,000 suspected cases were reported, and new introductions were recorded in neighboring Burundi, Rwanda, Kenya, and Uganda (*5*). In August 2024, a travel-associated clade Ib case was reported in Sweden (*6*). In response to the expanding burden of mpox in 2024, the Africa Centres for Disease Control and Prevention declared a public health emergency of continental security (*7*) and the World Health Organization declared a public health emergency of international concern (*4*).

Despite deployment of a modified vaccinia Ankara-Bavarian Nordic vaccine (https://www.bavarian-nordic.com) to many high- and middle-income countries, access mpox vaccine is not currently available for the general population in Africa. One vaccination model suggests that vaccination of 80% of children <15 years of age in DRC would result in robust reductions in illness, death, and MPXV circulation (*8*). We conducted a survey to determine population attitudes towards and willingness to receive mpox vaccination in DRC.

## The Study

For our cross-sectional analysis, we used data from a larger longitudinal telephone survey on COVID-19–related topics and vaccine hesitancy that began in 2022. The survey included participants from all 26 provinces in DRC and primarily targeted healthcare workers (HCWs). Participants were selected from historical telephone survey records of >10,000 persons. During December 2023–February 2024, trained interviewers contacted 5,226 adult participants and administered surveys in Swahili, French, Lingala, Kikongo, or Tshiluba. Survey questions addressed behavioral and social drivers of vaccination and attitudes toward introduction of new vaccines, including for mpox. Mpox vaccine acceptance was measured by asking participants about their interest in inclusion of an mpox vaccine in the national vaccination schedule. Response choices were yes, for adults, children, or both; no; and I do not know this disease (Appendix Table 1). We collapsed all yes responses for analysis. We assessed attitudes, vaccine acceptance by province, and sociodemographic characteristics via 5- or 3-level Likert scale questions and used a χ^2^ test for significance (α = 0.05). 

Among respondents, 79.2% were male, 20.8% were female, and most (12.6%) were from Kwilu Province. Participant representation from urban or rural settings was roughly equivalent. Most (41.2%) respondents reported some college or university education, and 55.5% identified as HCWs (Table).

**Table:** Characteristics of participants in a telephone phone survey about mpox vaccine acceptance conducted during December 2023–February 2024, Democratic Republic of the Congo*

Characteristics	Value, n = 5,226
Median age (IQR)	42 (26–58)
Sex	
M	4,138 (79.2)
F	1,088 (20.8)
Education level	
Less than high school	359 (6.9)
Graduated high school	1,589 (30.4)
Some college	2,158 (41.3)
Bachelor or advanced degree	1,120 (21.4)
Province	
Maindombe	134 (2.6)
Kwango	183 (3.5)
Kwilu	657 (12.6)
Kongo Central	312 (6.0)
Equateur	162 (3.1)
Mongala	238 (4.6)
Tshuapa	63 (1.2)
Nord Ubangi	113 (2.2)
Sud Ubangi	91 (1.7)
Kasai Oriental	597 (11.4)
Sankuru	176 (3.4)
Lomami	192 (3.7)
Kasai	118 (2.3)
Kasai Central	87 (1.7)
Haut Katanga	331 (6.3)
Tanganyika	151 (2.9)
Haut Lomami	50 (1.0)
Lualaba	168 (3.2)
Kinshasa	251 (4.8)
Maniema	98 (1.9)
North Kivu	140 (2.7)
Bas Uele	246 (4.7)
Haut Uele	137 (2.6)
Ituri	239 (4.6)
Tshopo	144 (2.8)
Sud Kivu	148 (2.8)
Geographic area	
Urban	2,659 (50.9)
Rural	2,567 (49.1)
Occupation	
Healthcare worker	2,899 (55.5)
Religious leader	89 (1.7)
Community or essential services leader	768 (14.7)
Public service sector or educator	670 (12.8)
Other worker	513 (9.8)
Not working	287 (5.5)
Health conditions	
Chronic lung disease	32 (0.6)
Diabetes	209 (4.0)
Cardiovascular disease	254 (4.9)
Chronic renal disease	12 (0.2)
Chronic liver disease	9 (0.2)
Immunocompromised	10 (0.2)
No conditions	4,700 (89.9)

*Values are no. (%) except as indicated. IQR, interquartile range.

**Figure 1:**
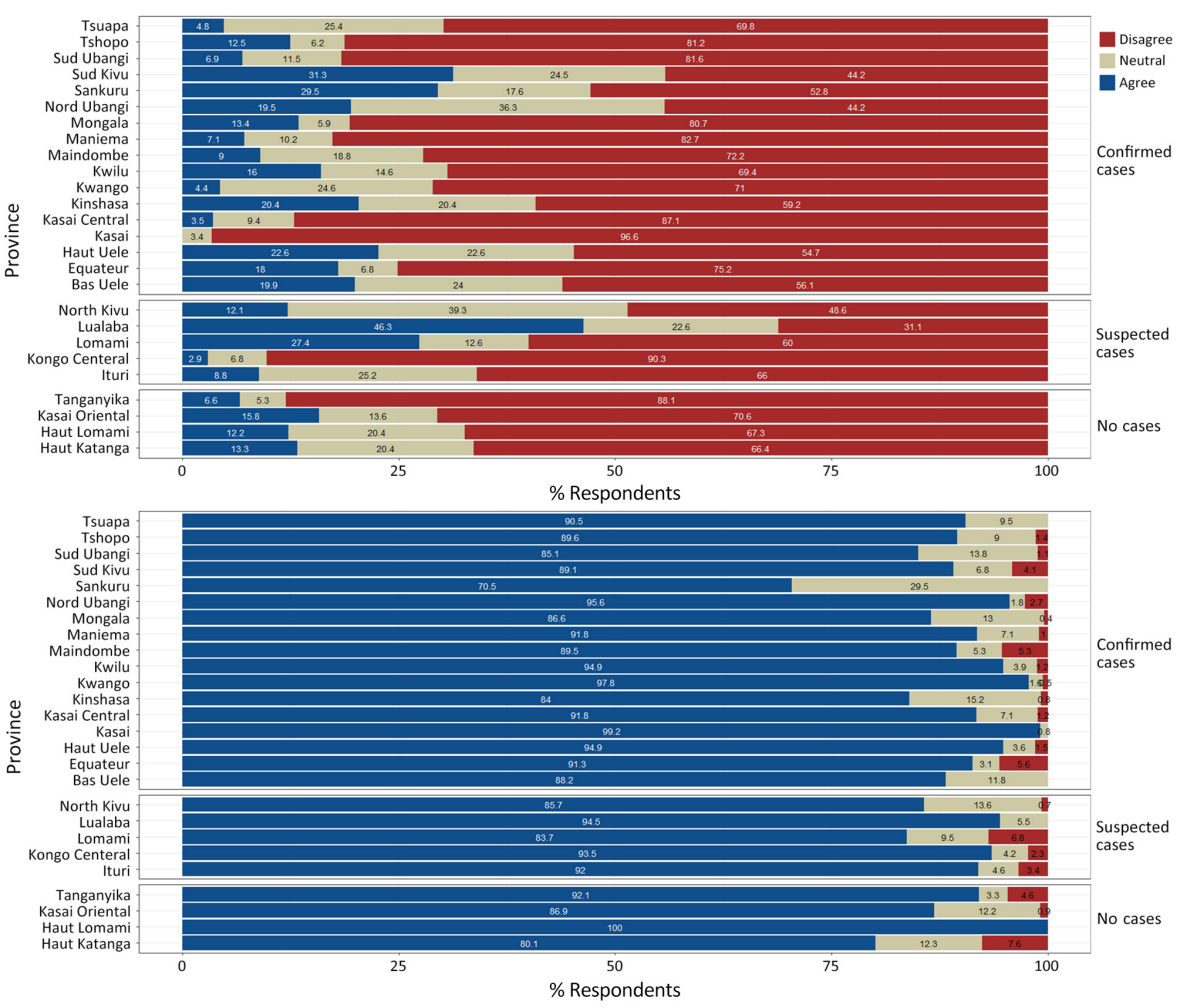
General mpox vaccine attitudes and perceptions from a telephone phone survey about mpox vaccine acceptance conducted during December 2023–February 2024, by province, Democratic Republic of the Congo. A) Reported responses to the statement: new vaccines carry more risks than older vaccines; B) reported responses to the statement: information I receive about vaccines from the vaccine program is reliable and trustworthy. Provinces are listed by whether they had confirmed, suspected, or no mpox cases detected. The 3-level responses were collapsed from a 5-point Likert scale.

Participants generally disagreed with the statement that new vaccines carry more risks than older vaccines. However, in 4 provinces, Sud Kivu, Nord Ubangi, Nord Kivu, and Lualaba, >50% of respondents agreed with or felt that greater risks exist with newer vaccines. Respondents generally agreed with the statement that information received from the vaccination program was reliable and trustworthy. In Sankuru Province, 29.5% of respondents felt neutral about the reliability of vaccine program information; the highest percentage of vaccine information distrust and disagreement with the statement were in Haut Katanga (7.6%) and Lomami (6.8%) Provinces (Figure 1; Appendix Table 2).

**Figure 2:**
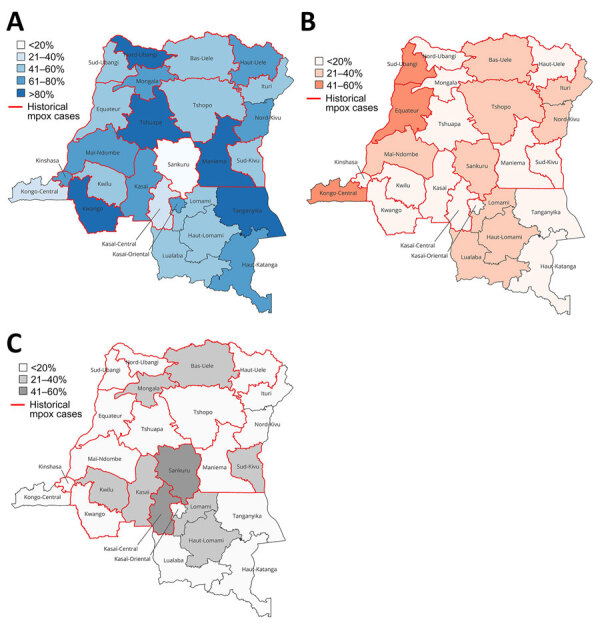
Geography of overall mpox vaccine acceptance perceptions from a telephone phone survey about mpox vaccine acceptance conducted during December 2023–February 2024, by province, Democratic Republic of the Congo. A) Reported acceptance collapsed from the following 3 responses: Yes, for all populations; Yes, for children only; and Yes, for adults only. B) Reported nonacceptance by the response to: No, not interested. C) Reported lack of knowledge of mpox disease. All cartographic figures generated by using QGIS version 3.36.3 (https://qgis.org). Percentages are reported by province. Red outlines indicate provinces with histories of mpox cases.

Nationally, 61.0% (95% CI 59.6%–62.4%) of respondents expressed acceptance of an mpox vaccination, 21.7% had no interest, and 17.3% reported no knowledge of mpox. At the province level, >80% of respondents from Kwango, Tshuapa, Nord Ubangi, Tanganyika, and Maniema reported vaccine acceptance. Sankuru had the lowest acceptance rates (19.9%; 95% CI 14.3%–26.6%) and highest percentage (57.4%; 95% CI 49.7%–64.8%) of participants reporting no mpox knowledge. In general, rates of mpox vaccine acceptance and mpox disease knowledge were not influenced by the history of mpox cases within the province (Figure 2).

**Figure 3:**
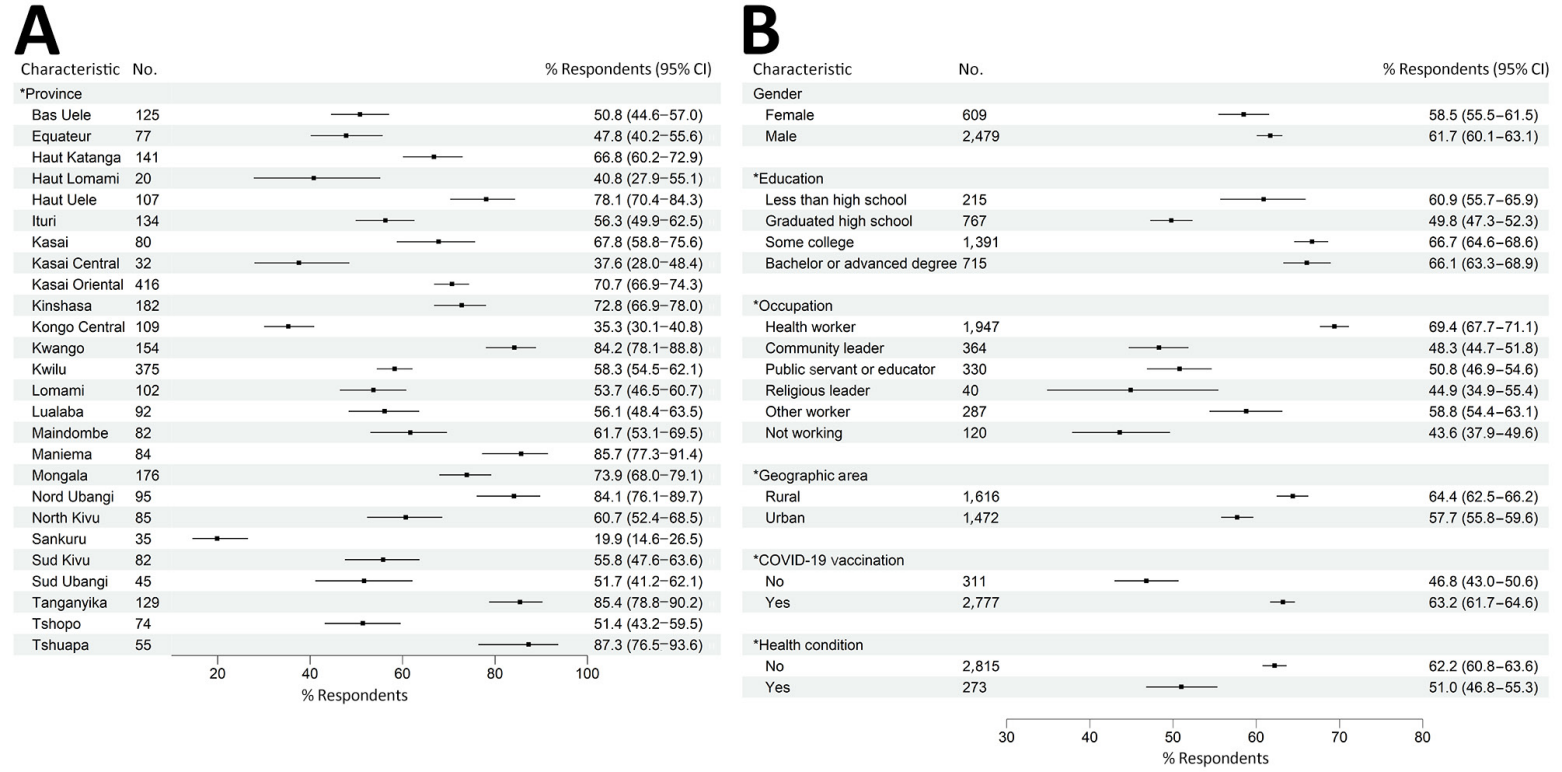
Percentage of mpox vaccine acceptance and demographic characteristic perceptions from a telephone phone survey about mpox vaccine acceptance conducted during December 2023–February 2024, by province, the Democratic Republic of the Congo. Responses were stratified by sociodemographic characteristics and known mpox risk factors; COVID-19 vaccination was dichotomized by whether the respondent received the COVID-19 vaccine. Health conditions were dichotomized on the basis of whether the respondent stated they had a chronic disease or were immunocompromised. Asterisks (*) indicate p<0.05 by χ^2^ test. Error bars indicate 95% CIs.

Interest in an mpox vaccine varied significantly by province (p<0.0001). When stratified by educational attainment, respondents with a high school diploma had much lower (49.8%; 95% CI 47.3%–52.3%) acceptance of the mpox vaccine than other groups. Among reported occupations, HCWs had the highest acceptance rates (69.4%; 95% CI 67.6%–71.1%). Respondents from rural locations indicated greater mpox vaccine acceptance than their urban counterparts (64.4% vs. 57.7%; p<0.0001). Among 4,396 respondents who reported receiving a COVID-19 vaccine, 63.2% indicated mpox vaccine acceptance. In addition, persons without chronic conditions reported higher rates of mpox vaccine acceptance than persons with chronic conditions (62.2% vs. 51.0%) (Figure 3).

## Conclusions

Given the ongoing mpox clade Ia and clade Ib outbreaks in the DRC and identification of cases in adjacent countries in Central Africa, introduction of a vaccine is increasingly needed. However, a paucity of information has been available regarding mpox knowledge and vaccine acceptance across DRC. We observed greater interest in mpox vaccine deployment among respondents from rural than urban locations. That finding could be explained by the historic mpox burden in DRC, where mpox cases primarily were among children in rural regions. Currently, reported mpox cases are rising in urban centers, further challenging response and vaccine planning efforts.

Assessing mpox vaccine trust and acceptance across provinces that have and have not experienced mpox cases and across demographic variables did not yield clear or neatly described trends. Instead, we observed several outliers that may reflect the diversity of DRC. For example, differing vaccine acceptance rates between persons with and without chronic health conditions may indicate underlying health anxieties among persons with persistent health problems; further investigation is needed to clarify that relationship. In addition, Sankuru Province was consistently a vaccine-hesitant outlier in this survey. Sankuru had the lowest percentage of respondents who trust information received from national vaccine programs and the lowest mpox vaccine acceptance, primarily because 57.4% of respondents stated no knowledge of mpox. In DRC, most mpox-endemic provinces consist of rainforest or forest-savanna mosaic geographies; the southeast provinces are primarily savanna and grassland and have no history of reported mpox cases. However, in June 2024, Lualaba Province in the southeast reported its first mpox case. Of note, that province had the highest percentage of respondents believing that new vaccines have greater risks than older vaccines, although overall trust in national vaccine programs was high.

Although our results report an overall trend of vaccine trust and acceptance nationwide, they also indicate the need for a multifaceted approach to vaccine education and rollout. Strategies should be tailored to historically endemic regions and areas with lower vaccine acceptance, such as Sankuru or Kasai Central, and consider how personal experiences and cultural considerations intersect with mpox outbreaks. Of note, Haut Lomami Province has been the setting of long-standing routine immunization revitalization efforts (*9*,*10*), and 100% of respondents from that province reported that they feel information from the national vaccine program is reliable. That highly positive response is perhaps reflective of the many years of vaccine campaigns and education efforts in the region.

This survey was limited to persons with access to telephones; persons with a lower socioeconomic status, including those in largely rural regions, may have been inadvertently excluded. This study included respondents from all provinces in DRC and targeted HCWs. Past studies in DRC have demonstrated 54% acceptance toward outbreak-related vaccines, and HCWs continually express the highest acceptance among respondents (*11*). Because this study sought to enroll HCWs and community and religious leaders, we observed an overrepresentation of men because men primarily hold leadership roles in the country. Because HCWs and religious leaders serve as health recommenders for their communities, however, identifying and addressing vaccine acceptance among those groups is critical. 

In conclusion, our findings provide insights regarding mpox vaccine acceptance within DRC. Our data highlight the need for increased community engagement and sensitization before widespread mpox vaccine deployment.

Appendix Additional information on mpox acceptance, Democratic Republic of the Congo.
